# Anesthetic Management for Emergent Repair of Tracheoinnominate Fistula

**DOI:** 10.1155/2020/8865303

**Published:** 2020-08-25

**Authors:** Vinayak Nadar, Ratan K. Banik

**Affiliations:** Department of Anesthesiology, School of Medicine, University of Minnesota and Fairview Medical Center, Minneapolis, MN, USA

## Abstract

We present a case of a 30-year-old female, who had tracheostomy revision complicated by false passage into the subcutaneous space and pneumothorax. Six days later, she developed massive bleeding from the mouth, nose, and tracheostomy site. Approximately 2 liters of blood was lost. With high suspicion for tracheo-innominate fistula, she was emergently brought to the operating room for fistula repair. Her anesthetic management was initially focused on maintaining spontaneous ventilation with inhalation agents until surgical exposure was adequate. An endotracheal tube was then placed under guidance of a video-laryngoscope. The tracheostomy tube was then removed over a Cook catheter to maintain secure passage in case of airway collapse. The oral endotracheal tube was then inserted distal to the arterial and tracheal defect. The patient's bleeding was stopped, the fistula was repaired, and she was transferred back to the intensive care unit, but she died several days later due to multi-organ failure.

## 1. Background

Tracheostomy is commonly performed in patients requiring long-term ventilator support (usually >2 weeks). The procedure is generally of low risk and is performed in the operative room or bedside. Tracheo-innominate fistula (TIF) is a rare complication of tracheostomy with an estimated incidence of 0.1% to 1% [1]. However, the complication can be highly lethal with mortality rates >75% [2, 3]. The cause of TIF is thought to be secondary to erosion from the tracheostomy tube through the tracheal wall into the innominate artery [1, 4]. Here, we report a case of TIF and discuss anesthetic management of TIF repair during massive hemorrhage through the fistula.

## 2. Case Report

A 30-year-old female with a past medical history including bipolar disorder, major depressive disorder, severe alcohol use disorder, alcoholic hepatitis, chronic pancreatitis, and gastric ulcers presented to an outside hospital with hematemesis and malaise. She was found to have acute alcoholic hepatitis, and treatment was started with supportive therapy, prednisone, and pentoxifylline. After failing to show signs of improvement, the patient was transferred to our hospital for extracorporeal liver assist device evaluation. Her Model for End-Stage Liver Disease score was 26. She had significant comorbidities including pancreatic insufficiency, candida esophagitis, and positive blood cultures for *Klebsiella*. Her condition continued to deteriorate until she developed acute respiratory failure with encephalopathy requiring endotracheal intubation and intensive care unit admission. A computed tomography scan was performed emergently, and it showed subarachnoid hemorrhage secondary to coagulopathy associated with her liver disease. She continued to have persistent muscular weakness and was unable to wean from the ventilator. After 2 weeks, she was scheduled for a bedside percutaneous tracheostomy.

The patient was stabilized hemodynamically but continued to have ongoing coagulopathy with thrombocytopenia, elevated INR, and PT. The initial tracheostomy placement was complicated by false passage with a left pneumothorax that required a chest tube placement. Eight days after the initial tracheostomy placement, a persistent cuff leak was noted and required emergency reintubation with an oral endotracheal tube. The patient then underwent a surgical tracheostomy revision complicated by a right-sided pneumothorax, which also required a chest tube placement.

Six days after her tracheostomy revision, she was noted to have massive bleeding from the tracheostomy site with severe occlusion of airway anatomy. Initially, bleeding was noted at her tracheostomy site. Pressure was held at the tracheostomy site to control bleeding, but then she was noted to have pooling of blood into the oropharynx and nose. There was a high suspicion of tracheo-innominate fistula.

Knowing the patient's ongoing coagulopathy with worsening INR and thrombocytopenia in the setting of acute bleeding, the patient was taken to the operating room (OR) emergently to control bleeding and secure the airway. The patient had an arterial line in place, and other standard ASA monitors were available continuously throughout the resuscitation. Our anesthetic management focused initially on verifying a patent airway despite profuse bleeding through the tracheostomy site while maintaining spontaneous ventilation with inhaled agents, small doses of midazolam/fentanyl, ketamine infusion and maintaining hemodynamics with transfusion of blood products and norepinephrine/vasopressin infusion.

Upon arrival to the OR, surgeons began exploring the tracheal site and found significant bleeding from the distal tracheal site, which was presumed to be from the innominate artery. The region was packed, and acute bleeding temporized. At this time, a bronchoscopy performed by the ENT surgery team through the tracheostomy site showed a right mainstem insertion of the tracheostomy tube. It was noted that the patient had a low tracheostomy site, being only 1.5 cm from the carina.

After further verifying that the majority of the bleeding was from the inferior region of the trachea, thoracic surgeons were called for help. It was not possible to control the bleeding through the neck incision, and it was noted that the posterior aspect of the artery was friable and lacerated.

Anticipating sternotomy and the need for a secured airway and use of paralytics, an oral endotracheal tube (ETT) was placed with aid of a video-laryngoscope just above the tracheostomy site. Then, a bronchoscope was passed through the oral ETT to visualize the distal trachea beyond the tracheal defect. At this point, a Cook catheter was placed through the tracheostomy tube and into the mainstem bronchus to allow a track in case of a lost airway situation. The ETT was then advanced under direct visualization into the distal trachea. ETT placement was confirmed under visualization with the bronchoscope, and then, the Cook catheter was removed.

The patient was paralyzed at this point. The surgeons performed a sternotomy for better exposure of the innominate artery. With the upper sternum open, surgeons dissected the anterior mediastinum towards the trachea and immediately noted that the bleeding was coming from a TIF fistula. The defect was a 5 × 2 mm hole in the innominate artery. It was repaired in a stepwise fashion so as to not fully cut off the blood supply to the distal innominate artery. At first, primary closure of the tracheal defect was attempted; however, it was unsuccessful. Therefore, the thoracic surgeons were once again involved, and they opened the abdomen to perform an omental flap for closure of the tracheal defect. For the rest of the surgery, the patient's airway was adequately maintained and she was transferred to the ICU intubated for further stabilization. Overall, the patient received 8 units of PRBC, 5 units of FFP, and 1 unit of platelets to address her bleeding and coagulopathy.

Postoperatively, the patient's clinical status remained tenuous with ongoing requirements for vasopressor support, mental status decline, and renal failure. The patient died several days later after a decision was made by the family to withdraw the ventilator and hemodynamic support.

## 3. Discussion

**Figure 1:**
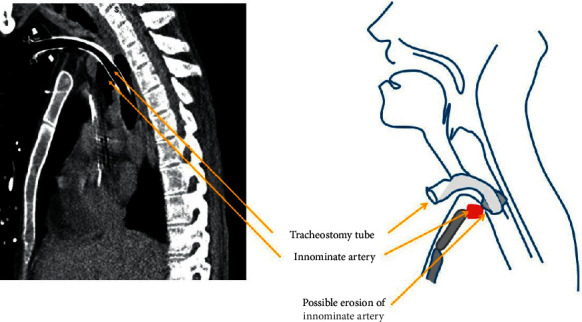
Pressure necrosis from tracheostomy cuff can erode innominate artery to form a trachea-innominate fistula. (a) Computed tomographic scan of this patient showing close proximity of the innominate artery to the tracheostomy tube. (b) Schematic diagram of the anatomical relationships in the formation of a fistula between the trachea and the innominate artery.

TIF is a rare complication due to an abnormal connection between the trachea and the brachiocephalic (innominate) artery [1, 4]. The main causes of this complication are low position of tracheostomy stoma, cuff overinflation, and infection [1]. Other risk factors include an extra-long tracheostomy cannula, patient with a short neck, difficulty in placement, fracture of tracheal cartilage, and localized inflammation [5–9]. Placement of an adequately sized cannula is important. The mechanical irritation from the cannula can possibly erode the tracheal wall, moving the cannula in close proximity to the innominate artery (Figure 1). Without early recognition and surgical management, TIF is fatal and carries a high mortality rate (>75%), even with intervention.

Whenever large, frank bleeding is noted from the tracheostomy site, and the patient should be brought to OR without delay. Aspiration of blood, visual pulsation of the cannula, and naso-oropharyngeal hemorrhage are clear signs, but they are not always present. It is prudent not to spend valuable time on bedside fiber-optic bronchoscopy or angiography to confirm diagnosis because massive hemorrhage and hemodynamic collapse can follow at any time after the initial presentation.

The priorities of the anesthesiologist are to control the airway, maintain spontaneous ventilation (airway collapse can occur due to massive hemorrhages), control bleeding, initiate a massive transfusion protocol, and involve a multidisciplinary surgery team including thoracic, ENT, and general surgeons for repair of the injured artery and tracheal defect. An attempt to tamponade the bleeding site should be made by immediate overinflation of the tracheostomy tube balloon, with simultaneous retraction of the tube anteriorly. Such a procedure can be both therapeutic and diagnostic, but it is only a temporizing measure to buy time for operative intervention.

If the bleeding is stopped, blood must be aspirated from the airway below and an endotracheal tube should be placed, with the balloon more distal than the tracheostomy tube. If oral intubation is difficult due to massive hemorrhage, passage of a small-sized oral ETT or pediatric endotracheal tube through the patient's tracheostomy cannula can be attempted. It is also recommended that a Cook catheter is placed into the existing tracheostomy site prior to the removal of tracheostomy tube to ensure a patent passage in case of airway collapse. This method of securing the existing tracheostomy passage can be critical in a situation where there is acute bleeding and is easy to overlook during a fast-moving emergency.

TIF is an extremely rare life-threatening complication with a high mortality rate [1, 4]. The prompt diagnosis and emergency measures are the key to the patient's survival. The role of anesthesiologists as part of a multidisciplinary team is extremely important and is outlined in the following.

### 3.1. Clinical Pearls

1. Call for help immediately, activate massive transfusion protocol, notify ENT/thoracic/vascular surgery. Maintain external pressure into the stoma and transport the patient to the OR emergently for surgical management.
2. Evaluate the ability to ventilate; ensure that suction and emergency airway equipment are available.
3. If able to ventilate, discuss with an ENT surgeon about possible early intervention. Consider introducing air slowly into the tracheostomy cuff (slow hyperinflation) and reduce ongoing bleeding by using direct pressure at the stoma site or pressure at the sternal notch.
4. If unable to ventilate, there is likely bleeding deep from the stoma site and the patient will need a new airway. Attempt to intubate with a cuffed ETT either orally or through the stoma. If oral intubation is considered, use a long ETT with deep insertion to bypass the bleeding site.
5. Consider a Cook/exchange catheter prior to removal of tracheostomy tube to ensure quick reinsertion via a secure passage if tracheal occlusion occurs distal to the oral ETT site. Massive bleeding can quickly distort anatomy.

## Data Availability

No data were used to support this study.
